# Maternal smoking early in pregnancy is associated with increased risk of short stature and obesity in adult daughters

**DOI:** 10.1038/s41598-019-39006-7

**Published:** 2019-03-12

**Authors:** Sarah E. Maessen, Fredrik Ahlsson, Maria Lundgren, Wayne S. Cutfield, José G. B. Derraik

**Affiliations:** 1 Liggins Institute, University of Auckland, Auckland, New Zealand; 2 Department of Women’s and Children’s Health, Uppsala University, Uppsala, Sweden; 3 A Better Start – National Science Challenge, University of Auckland, Auckland, New Zealand

## Abstract

We assessed anthropometry in 22,421 adult daughters in association with their mothers’ tobacco smoking early in pregnancy (at their first antenatal visit) in Sweden, particularly their risk of short stature and obesity. Adult daughters were grouped by maternal smoking levels during pregnancy: Non-smokers (58.5%), Light smokers (24.1%; smoked 1–9 cigarettes/day), and Heavier smokers (17.4%; smoked ≥10 cigarettes/day). Anthropometry was recorded on the adult daughters at approximately 26.0 years of age. Obesity was defined as BMI ≥30 kg/m^2^, and short stature as height more than two standard deviations below the population mean. Daughters whose mothers were Light and Heavier smokers in early pregnancy were 0.8 cm and 1.0 cm shorter, 2.3 kg and 2.6 kg heavier, and had BMI 0.84 kg/m^2^ and 1.15 kg/m^2^ greater, respectively, than daughters of Non-smokers. The adjusted relative risk of short stature was 55% higher in women born to smokers, irrespectively of smoking levels. Maternal smoking had a dose-dependent association with obesity risk, with offspring of Heavier smokers 61% and of Light smokers 37% more likely to be obese than the daughters of Non-smokers. In conclusion, maternal smoking in pregnancy was associated with an increased risk of short stature and obesity in their adult daughters.

## Introduction

The harmful effects of smoking are widely established. For pregnant women, these effects are known to extend to the fetus, leading to a variety of adverse pre- and post-natal health effects^1^. Trends in tobacco use vary between nations and age groups, but in general the number of people in developed nations who smoke has declined steadily over the last few decades^2^. This decline appears to be slower for younger women than other groups^3^, and importantly, women who smoke do not necessarily quit if they become pregnant^4,5^.

It is estimated that 5.5 to 7% of Swedish women currently smoke in early pregnancy, while approximately 25% smoke prior to pregnancy^6,7^. The number is much higher for teenage mothers in Sweden, of whom one in four smoke during pregnancy^7^. In New Zealand in 2015, 14.2% of women reported smoking in early pregnancy^8^. However, the actual proportion of women who smoke during pregnancy in both Sweden and New Zealand is likely to be higher than these estimates based on self-report. When compared to biological markers of smoking, self-reports have significantly underestimated both the number of women who smoke during pregnancy and the number of cigarettes they smoke in these two countries^9,10^.

Smoking during pregnancy increases the risk of pregnancy complications, birth defects, and miscarriage^1,11^. It is associated with an increased risk of sudden death in early life^12^, lower birth weight^13–17^, poorer newborn lung function^18^, and increased risk of asthma in childhood^19^.

The newborns whose mothers smoked during pregnancy are usually reported to be proportionally smaller on average, i.e. they are shorter at birth as well as weighing less than other infants^16,20,21^. Children exposed to tobacco *in utero* may remain up to a centimeter shorter on average than their peers in childhood^16,20,22^, but other studies have not observed an effect on stature^23,24^. The British longitudinal National Child Development Study (NCDS) reported that women who smoked more than 20 cigarettes per day during pregnancy in 1958 had offspring who were more than a centimeter shorter in adulthood than those whose mothers did not smoke^25^.

Importantly, children born to mothers who smoked during pregnancy have an increased risk of being obese^21,26–30^. The association between maternal smoking in pregnancy and obesity in the offspring may persist into adulthood; the NCDS demonstrated that offspring of British women who were pregnant in 1958 had an increased risk of obesity throughout childhood and into adulthood if their mothers smoked during late pregnancy^31^. In a more recent cohort, Mattsson *et al*. described a dose-dependent effect of maternal cigarette smoking during early pregnancy on obesity risk in Swedish women^32^. However, that study did not control for maternal anthropometry, which is strongly associated with offspring anthropometry at birth^33^ and in adulthood^34^.

Due to the changing rates of both smoking and obesity, it cannot be assumed that results of the NCDS are applicable to a younger cohort, or to the offspring of women who smoked earlier in pregnancy. In addition, little is known about the long-term effects of light or moderate maternal smoking during pregnancy on adult height. Thus, in the present study we aimed the examine the association between maternal smoking and adult anthropometry (particularly on the risks of obesity and short stature) in a large cohort of women born to mothers who smoked early in pregnancy (first trimester) between 1973 and 1988 in Sweden, while also accounting for the effects of maternal anthropometry.

## Methods

### Ethics

Ethics approval was obtained from Uppsala’s Regional Ethical Review Board. This investigation was carried out in accordance with approved national and international guidelines for medical research. This is a register-based study on anonymized data where participants were not contacted; thus, informed consent was not required.

### Study design

Data were extracted from the Swedish Birth Register, which records information on >99% of all births in Sweden, with a low error rate for key parameters when compared to the original medical records^35^. Data are prospectively recorded in pregnancy starting at the first antenatal visit and then forwarded to the Birth Register. In this study, the data recorded at the mother’s first antenatal visit were linked to the medical records on their daughter’s own subsequent first antenatal visit.

Our study covers from the first antenatal visit (mostly 10–12 weeks of gestation) on 303,301 women born in 1973–1988 in Sweden, who gave birth in 1991–2009 and were aged ≥18 years. Exclusion criteria were non-Nordic ethnicity; extremely short stature (≤130 cm); being born preterm (<37 weeks of gestation)^36^, post-term (≥42 weeks of gestation)^37^, or <−3 standard deviation scores (SDS) below the Swedish population mean for birth weight and/or birth length^38^; or having congenital malformations (ICD-9 740–759 and ICD-10 Q0–Q99).

Weight and height were measured, although height was self-reported in some cases. Gestational age at their birth (extracted from the Swedish Birth Register) was estimated from the date of the last menstrual period for the majority of participants, otherwise estimates were based on ultrasound scans. Overweight was defined as body mass index (BMI) ≥25 kg/m^2^ and <30 kg/m^2^, and obese as ≥30 kg/m^2^. Short stature was defined as height >2 SDS below the study population mean (<155 cm). Ponderal index (g/cm^3^) was calculated as per Röhrer’s formula [(100 x weight)/length].

### Statistical analyses

Stratified analyses were carried out to examine differences in outcomes between daughters of smokers vs non-smokers, with the latter group also split into Light smokers (reported smoking between one and nine cigarettes per day) and Heavier smokers (10 or more cigarettes daily). Parameters at birth were compared between groups using univariable general linear regression models. Adult anthropometry, risk of obesity, and risk of short stature were examined with generalized linear regression models. The adjusted models included the following variables: birth order, age, and year of birth (to account for population-wide trends); in addition, models on daughter’s weight, BMI, and overweight/obesity risk adjusted for their current smoking habits, as well as their mothers’ weight or BMI; while the models on women’s stature were also adjusted for maternal height.

Binary outcomes are expressed as adjusted relative risks (aRR) and respective 95% confidence intervals. Analyses were performed in SAS v9.4 (SAS Institute, Cary, USA) and SPSS v24 (IBM Corp, Armonk, NY, USA). All tests were two-tailed, without adjustment for multiple comparisons.

## Results

**Figure 1:**
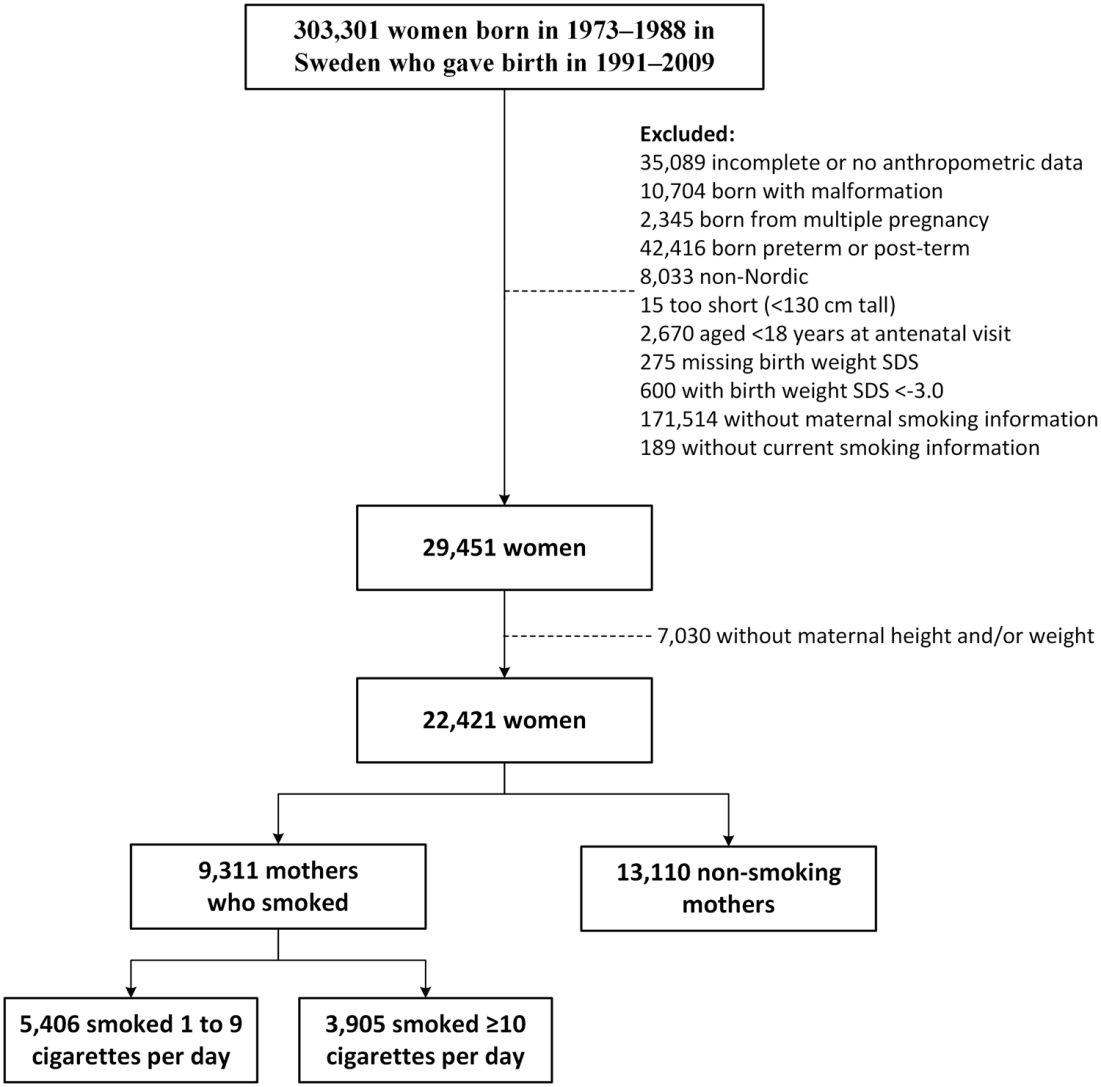
Diagram showing the inclusion and exclusion of participants with data extracted from the Swedish Birth Register.

From the original sample of 303,301 women, 22,421 met the inclusion criteria and had recorded information on the mother’s BMI and smoking status during pregnancy; 9,311 mothers (41.5%) had reported they were smoking at their first antenatal visit (Fig. 1). From this group of women, 5,406 had mothers (24.1% overall) who had been Light smokers and 3,905 (17.4% overall) Heavier smokers during pregnancy. Of note, the demographic and anthropometric characteristics of the 22,421 women included in this study were similar to those who were excluded (Supplementary Table 1).

### At birth

Women born to mothers who smoked in early pregnancy, on average, weighed 208 g less, were 8 mm shorter, and had a ponderal index that was 0.03 g/cm^3^ lower than those born of Non-smokers (Table 1). There was a dose-dependent relationship between maternal smoking level and the daughter’s weight and length at birth, with Heavier smokers giving birth to daughters 241 g lighter and 10 mm shorter than Non-smokers on average (Table 1).

**Table 1:** Parameters recorded at birth for 22,421 women born in Sweden in 1973–1988 according to their mothers’ smoking status and level of smoking early in pregnancy.

	Mother Non-smoker	Mother Smoker	Mother Light Smoker	Mother Heavier Smoker
n (%)	13,110 (58.5%)	9,311 (41.5%)	5,406 (24.1%)	3,905 (17.4%)
Birth weight (g)	3,547 (3,539, 3,555)	3,339 (3,330, 3,349)****	3,364 (3,352–3,376)****	3,306 (3,291, 3,320)****^††††^
Birth weight SDS	0.43 (0.41, 0.44)	−0.02 (−0.04, 0.00)****	0.03 (0.00–0.05)****	−0.09 (−0.12, −0.06)****^††††^
Birth length (cm)	50.3 (50.3, 50.3)	49.5 (49.4, 49.5)****	49.6 (49.6–49.7)****	49.3 (49.3, 49.4)****^††††^
Birth length SDS	0.40 (0.39, 0.42)	−0.03 (−0.05, −0.01)****	0.03 (0.01–0.06)****	−0.11 (−0.14, −0.08)****^††††^
Ponderal index (g/cm^3^)	2.78 (2.77, 2.78)	2.75 (2.75, 2.75)****	2.75 (2.74–2.75)****	2.75 (2.74, 2.76)****
Gestational age (weeks)	39.5 (39.5, 39.5)	39.4 (39.4, 39.4)****	39.4 (39.4, 39.5)****	39.4 (39.4, 39.4)****

SDS, standard deviation scores.

^****^p < 0.0001 for comparisons to daughters of Non-smokers.

^††††^p < 0.0001 for comparisons between daughters of Light and Heavier smokers.

Light smoker was defined as smoking 1 to 9 cigarettes per day, and Heavier smoker as ≥ 10 cigarettes per day. Data are means and 95% confidence intervals.

### Adult anthropometry

Women born to mothers who smoked were 1.1 cm shorter and 1.5 kg heavier on average in adulthood than those whose mothers did not smoke early in pregnancy, with an average BMI that was 0.84 kg/m^2^ higher (Table 2). There was a dose-dependent association with body mass (but not with height), so that women born to mothers who were Heavier smokers were 1.9 kg heavier and had a BMI that was 1.04 kg/m^2^ greater than the offspring of Non-smokers (Table 2).

**Table 2:** Anthropometric data recorded early in pregnancy (mostly 10–12 weeks) in 1991–2009 among 22,421 women who were born at term in Sweden in 1973–1988 according to their mothers’ level of smoking early in pregnancy.

		Mother Non-smoker	Mother Smoker	Mother Light Smoker	Mother Heavier Smoker
n (%)		13,110 (58.5%)	9,311 (41.5%)	5,406 (24.1%)	3,905 (17.4%)
Age (years)		26.3 ± 5.0	25.5 ± 5.1****	25.2 ± 4.9****	26.0 ± 5.2**** ^††††^
Unadjusted data	**Height (cm)**	167.1 (167.0, 167.2)	166.0 (165.9, 166.1)****	166.1 (166.0, 166.3)****	165.9 (165.7, 166.1)****
	**Weight (kg)**	67.26 (67.02, 67.50)	68.75 (68.46, 69.04)****	68.43 (68.06, 68.81)****	69.19 (68.75, 69.63)**** ^†††^
	**BMI (kg/m**^2^)	24.07 (23.99, 24.15)	24.91 (24.81, 25.01)****	24.76 (24.63, 24.89)****	25.11 (24.96, 25.26)**** ^††††^
BMI status	**Underweight**	528 (4.0%)	325 (3.5%)	183 (3.4%)	142 (3.6%)
	**Normal weight**	8,367 (63.8%)	5,368 (57.7%)	3,188 (59.0%)	2,180 (55.8%)
	**Overweight**	2,872 (21.9%)	2,205 (23.7%)	1,281 (23.7%)	924 (23.7%)
	**Obese**	1,343 (10.2%)	1,413 (15.2%)	754 (13.9%)	859 (16.9%)
	**Overweight/obese**	4,215 (32.2%)	3,618 (38.9%)	2,035 (37.6%)	1,583 (40.5%)
Short stature		189 (1.4%)	208 (2.2%)	122 (2.3%)	86 (2.2%)
Adjusted data	**Height (cm)**	167.0 (166.9, 167.1)	166.1 (166.0, 166.2)****	166.2 (166.1, 166.3)****	166.0 (165.8, 166.1)**** ^†^
	**Weight (kg)**	66.94 (66.72, 67.16)	69.50 (69.25, 69.75)****	69.24 (68.91, 69.56)****	69.87 (69.48, 70.25)**** ^†^
	**BMI (kg/m**^2^)	24.07 (23.99, 24.15)	25.04 (24.95, 25.13)****	24.91 (24.79, 25.03)****	25.22 (25.08, 25.37)**** ^††^

Underweight BMI <18.5 kg/m^2^; normal weight ≥18.5 kg/m^2^ and <25 kg/m^2^; overweight ≥25 kg/m^2^ and <30 kg/m^2^; overweight/obese ≥25 kg/m^2^; and obese ≥30 kg/m^2^.

Short stature defined as height more than 2 standard deviations below the population mean (i.e. <155 cm).

Adjusted data were analysed using generalized linear regression models, adjusting for birth order, age, and year of birth (to account for population-wide trends); in addition, models on women’s weight and BMI adjusted for their current smoking habits, as well as their mothers’ weight or BMI, respectively; while the model on women’s height was also adjusted for their mothers’ height.

^****^p < 0.0001 for comparisons to daughters of Non-smokers.

^†^p < 0.05, ^††^p < 0.01, ^†††^p < 0.001, and ^††††^p < 0.0001 for comparisons between daughters of Light and Heavier smokers.

Light smoker was defined as smoking 1 to 9 cigarettes per day, and Heavier smoker as ≥10 cigarettes per day.

Age data are means ± standard deviations; categorical data are n (%); other data are means and 95% confidence intervals.

The association between maternal smoking in early pregnancy and daughters’ heights in adulthood was slightly altered after adjustment for confounding factors, with evidence of a subtle dose-dependent relationship (Table 2). Women born to Light and Heavier smokers were 0.8 and 1.0 cm shorter, respectively, than those born to Non-smokers (Table 2).

In contrast, the possible effects of maternal smoking during pregnancy were accentuated for body mass (Table 2). Compared to daughters of mothers who did not smoke, those whose mothers were Light and Heavier smokers were 2.3 and 2.9 kg heavier, respectively, with BMI that was 0.84 and 1.15 kg/m^2^ greater (Table 2).

### Risk of short stature

Rates of short stature were higher in daughters of smoking mothers, irrespective of smoking levels (Table 2). Women whose mothers smoked during pregnancy were 55% more likely to have short stature in adulthood (Table 3). However, unlike the obesity risk, the likelihood of short stature did not appear to be associated with the level of maternal smoking in early pregnancy (Table 3).

**Table 3:** Unadjusted and adjusted relative risks (RR) of adult short stature among 22,421 Swedish women born at term according to their mothers’ level of smoking early in pregnancy.

Maternal smoking levels	Unadjusted RR	*p*	Adjusted RR	*p*
All smokers vs Non-smokers	1.55 (1.27, 1.88)	<0.0001	1.55 (1.28, 1.88)	<0.0001
Light smokers vs Non-smokers	1.57 (1.25, 1.96)	<0.0001	1.55 (1.24, 1.93)	<0.001
Heavy smokers vs Non-smokers	1.53 (1.19, 1.97)	0.001	1.57 (1.23, 2.00)	<0.001
Heavy smokers vs Light smokers	0.98 (0.74, 1.28)	0.861	1.01 (0.78, 1.31)	0.925

Women were born in Sweden in 1973–1988, and body mass index (BMI) data were recorded in 1991–2009 at a mean age of 26.0 years. Data were analysed using generalized linear regression models. Adjusted relative risks accounted for maternal height, birth order, age, and year of birth. Short stature is defined as height >2 standand deviations below the population mean (i.e. <155 cm), Light smoker as smoking 1 to 9 cigarettes per day, and Heavier smoker ≥10 cigarettes per day.

### Obesity risk

A higher proportion of women whose mothers smoked during pregnancy were overweight and/or obese as adults than the daughters of Non-smokers (Table 2). Obesity rates increased with smoking levels, from 10.2% amongst daughters of Non-smokers to 13.9% and 16.9% in those born to Light and Heavier smokers, respectively (Table 2).

The adjusted relative risk (aRR) of obesity for women whose mothers smoked during pregnancy was 1.47 times higher than for daughters whose mothers were Non-smokers (Table 4). There was a dose-dependent association with obesity risk, which was 1.37 for women born to Light smokers and 1.61 for those born to Heavier smokers (Table 4).

**Table 4:** Unadjusted and adjusted relative risks (RR) of obesity early in pregnancy among 22,421 Swedish women born at term according to their mothers’ level of smoking early in pregnancy.

Maternal smoking levels	Unadjusted RR	*p*	Adjusted RR	*p*
All smokers vs Non-smokers	1.48 (1.38, 1.59)	<0.0001	1.47 (1.37, 1.58)	<0.0001
Light smokers vs Non-smokers	1.36 (1.25, 1.48)	<0.0001	1.37 (1.26, 1.49)	<0.0001
Heavier smokers vs Non-smokers	1.65 (1.51, 1.80)	<0.0001	1.61 (1.48, 1.76)	<0.0001
Heavier smokers vs Light smokers	1.21 (1.10, 1.33)	<0.001	1.18 (1.08, 1.29)	<0.001

Women were born in Sweden in 1973–1988, and body mass index (BMI) data were recorded in 1991–2009 at a mean age of 26.0 years. Data were analysed using generalized linear regression models. Adjusted relative risks accounted for maternal BMI, birth order, age, year of birth, and current regular smoking. Obesity is defined as BMI ≥30 kg/m^2^, Light smoker as smoking 1 to 9 cigarettes per day, and Heavier smoker ≥10 cigarettes per day.

### Sensitivity analyses

Exploratory analyses were carried out without the exclusion of women who were born preterm or post-term. The associations between maternal smoking and anthropometric measurements on adult daughters were unchanged when compared to the findings for just those who were born at term (Supplementary Table 2). The risk of obesity in association with maternal smoking was also unchanged (Supplementary Table 3). However, in contrast to the findings on women born at term, there was some evidence of a dose-dependent effect of maternal smoking on their daughters’ stature, with the adjusted relative risk increasing from 1.48 in those born to Light smokers to 1.63 among daughters of Heavier smokers (Supplementary Table 3).

Of note, our data suggest that the association with a risk of obesity in adulthood was stronger with maternal smoking than with the daughters’ current smoking habits (Supplementary Table 4). Further, there was also evidence that maternal smoking was an important factor for the development of a smoking habit in their daughters [aRR 1.43 (95% CI 1.38, 1.47); p < 0.0001].

## Discussion

Our study showed that maternal smoking in early pregnancy was associated with increased risk of short stature (+55%) in the adult daughters and a marked dose-dependent relationship with obesity risk. Women whose mothers smoked in early pregnancy were approximately 1 cm shorter, while daughters born to Heavier smokers (≥10 cigarettes per day) were on average 2.9 kg heavier with an adjusted relative risk of obesity that was 61% higher. In addition, consistent with previous research, women in our study whose mothers smoked during early pregnancy were lighter and shorter at birth than offspring of non-smokers.

This is the first study to report the long-term associations between smoking in early pregnancy and adult height, and one of few to investigate associations between maternal smoking at any stage of pregnancy with long-term outcomes. Similarly to our findings for mothers who smoked in early pregnancy, the smaller 1958 National Child Development Study (NCDS) reported an increased risk of obesity in adulthood for men and women (44% for the latter) whose mothers smoked beyond the fourth month of pregnancy, after controlling for maternal and environmental factors^31^. Previously, Mattsson *et al*. showed a very similar dose-dependent association between cigarette smoking in early pregnancy and offspring obesity risk in Swedish women^32^. Our study has strengthened those findings, as we have confirmed that the association between maternal smoking during pregnancy with an increased risk of obesity in the adult offspring not only remains after adjustment for maternal anthropometry, but that the apparent effects on weight and BMI become more pronounced. In addition, we also showed a novel association between maternal smoking during pregnancy and an increased risk of short stature in the offspring, irrespective of smoking levels.

Longitudinal studies suggest that the risk of obesity in children born to mothers who smoked during pregnancy increases as they get older^13,15,26^. The results of our study confirm previous research reporting that the increased risk of obesity for offspring of women who smoke while pregnant persists into adulthood^31,32^, although the relationship may not be as strong as in childhood. Children whose mothers smoked during pregnancy are typically estimated to be between 1.7 and 3.4 times more likely to be obese than other children^15,21,28–30^, including studies where smoking status was assessed early in pregnancy^15,21^. In comparison, maternal overweight and obesity have been estimated to increase the odds of offspring obesity in adulthood more than two-fold^39^. Due to the complex combination of shared genetic and environmental influences involved in this relationship, little is known about whether interventions focused on maternal weight loss are effective in reducing the obesity risk for offspring^39,40^. However, there is some evidence that smoking cessation during pregnancy can reduce the risk of low infant birthweight^14^ and subsequent obesity risk^31^.

No studies of adult outcomes have compared maternal smoking in early pregnancy vs late in pregnancy, but a German study reported that the risk of childhood obesity may be similar between offspring of women who quit smoking early in pregnancy and those whose mothers continued smoking throughout pregnancy^27^. In contrast, it seems that the relationship between maternal smoking and lower birthweight appears to be strongest when smoking continues into the third trimester^41^, the time when most fetal growth occurs. In our study, we were not able to identify the mothers who smoked throughout pregnancy and those who smoked only in early pregnancy. Nonetheless, the similarity of our results to the NCDS suggest that smoking in early pregnancy (irrespective of continuous smoking further into pregnancy) influences the future obesity risk of the offspring.

To our knowledge, the NCDS study is the only other to have examined the association between maternal smoking in pregnancy and adult height^25^. Consistent with our findings, women in the NCDS whose mothers had smoked during pregnancy were approximately 1 cm shorter than those born to non-smokers^25^. However, this association was observed only for very heavy smokers (smoking more than 20 cigarettes per day)^25^. Further, the association was no longer statistically significant after controlling for confounding factors^25^. Women born to both Light and Heavier smokers in our study had a markedly increased risk of short stature than offspring of non-smokers, and the relationships persisted after adjustment for confounding factors (including maternal anthropometry). Nonetheless, in contrast to our observations, studies on pre-pubertal children and teenagers have suggested a dose-dependent association between maternal smoking and stature, though the height differences between offspring of light smokers and heavy smokers was small (0.1 to 0.2 cm)^22,42^. Overall, the observed risk of short stature in association with maternal smoking during pregnancy is of similar magnitude (although slightly higher) to that observed in Swedish women born moderately preterm^36^. Further, we observed that average adult height in women born to Heavier smokers was 1 cm shorter than the offspring of non-smokers, while in comparison, women who were born during the Chinese Famine (1959–1961) were 1.7 cm shorter as adults^43^.

Although the mean difference in height between daughters of smokers and non-smokers was small at the individual level in comparison to other determinants of adult height, we reported an increased risk of short stature for daughters whose mothers smoked in early pregnancy. Shorter stature has been related to poorer social outcomes, such as a reduced likelihood of obtaining tertiary educational qualifications and increased suicide risk^44,45^. Height is also associated with many markers of cardiovascular health, with shorter stature relating to greater non-HDL cholesterol and triglycerides^46^, higher systolic and diastolic blood pressure^46,47^, as well as an increased risk of coronary artery disease^46,48,49^ and stroke^49^. The relationship between height and cardiovascular risk factors appears to be linear, with the greatest risk among the shortest members of society^50^. Although some health outcomes (such as lung function) can be directly affected by shorter height due to mechanical constraints^46^, it is unclear whether short stature itself is disadvantageous for cardiovascular health or is simply a marker of early growth and development.

Smoking during pregnancy has consistently been associated with decreased growth *in utero*. On average, mothers who smoke have infants with lower birth weight than those born to non-smokers^13–17^. This relationship seems to be explained in part by a reduction in insulin-like growth factor 1 (IGF-1, a key driver of fetal growth) levels, as observed in cord blood of fetuses exposed to maternal tobacco smoking^17^. In general, IGF-1 concentrations are influenced by placental nutrient supply, and the nicotine and carbon monoxide exposure associated with smoking during pregnancy reduce both the blood flow to the fetus and its oxygen content^51^.

Although children born to mothers who smoked during pregnancy are usually smaller^13–17^, they tend to experience a period of rapid growth post-natally^15,16^, and are likely to catch up to their peers in height and weight as early as within the first 12 months of life^52^. Following this catch-up period, children of mothers who smoked during pregnancy continue to grow at a faster rate than their peers, with proportionally higher increases in fat mass^15^. This pattern of growth is common to other populations of infants with low birthweight^53,54^. Catch-up growth is thought to provide benefits in the short term, but poses risks to health in the long term^54^.

By five years of age, children born to smokers are around twice as likely to be obese compared to the offspring of non-smoking mothers^21,26–30^. This later weight gain is thought to be a result of fetal programming, where the fetus has adapted to poor nutrient supply *in utero*, resulting in permanent changes to metabolism that are maladaptive later in life^55^. A high BMI following low birth weight has been associated with more health risks than a high BMI with high birth weight^56^. Thus, the apparent effects of maternal smoking on fetal and infant growth could be underpinning the adverse long-term associations we and others have observed.

Of note, several recent studies have explored the role of epigenetics as a pathway by which maternal smoking in pregnancy affects the future health of their offspring. A number of studies have compared the offspring of mothers who did or did not smoke during pregnancy, and cord or newborn heel-prick blood revealed differential methylation of genes known to be involved in the response to tobacco-related compounds^57,58^, embryonic and fetal development^58,59^, and immune system function^58,60^. At least one study has reported that differential methylation of specific genes partially mediates the relationship between maternal smoking in pregnancy and their offspring’s birth weight^60^. However, there is still much to be learnt about how epigenetics may influence the effects of maternal smoking on offspring health. For example, a meta-analysis identified nearly 3000 sites corresponding to genes whose expression in newborns appeared to be affected by maternal smoking during pregnancy^57^.

Our study has some limitations. Data about smoking status was not consistently collected when the participants’ mothers were pregnant, so our sample was not randomly selected and cannot be assumed to be the prevalence of smoking during pregnancy at the time. Importantly, smoking data were not collected later in pregnancy beyond the first antenatal visit, so it is not known how many mothers continued to smoke throughout pregnancy, nor can we compare the effects of smoking throughout pregnancy with early pregnancy alone. A Swedish study on women who were pregnant on 1988 (overlapping with the time when mothers of our participants were pregnant) reported that only 18% of smokers quit prior to their first antenatal visit, with 65% of women continuing to smoke throughout pregnancy^4^. Based on this information and the similarity of our findings to those reported by the NCDS (where smoking status was assessed later in pregnancy), it is likely that the majority of mothers in our study who were smoking at the first antenatal visit continued to smoke throughout pregnancy. Anthropometry of the daughters in our study was also assessed in early pregnancy. While data based on self-reported pre-pregnancy weight indicated that women gained an average of 4 kg in the first 16 weeks of pregnancy^61^, there is no evidence to suggest that early pregnancy weight gain would differ between women whose mothers smoked during pregnancy and those whose mothers were non-smokers. Therefore, this previously observed weight gain is unlikely to affect the associations between maternal smoking and daughter’s obesity risk described in our study. However, the mean weight and BMI data reported here might have overestimated the obesity risk for some women in both groups. Lastly, we did not have data on maternal second-hand smoking during pregnancy, socioeconomic status, or lifestyle factors (e.g. diet and physical activity) that could have affected our results.

However, our study has important strengths. We studied a large and relatively homogenous cohort of Nordic women. In addition, we have not only accounted for the important confounding effects of maternal anthropometry in our statistical models, but we have also excluded important groups with an increased risk of short stature such as women born preterm^36^ or those of extremely low birth weight^62^.

Overall, our results emphasize the importance of early intervention for women who smoke to reduce the long-term health risks to their offspring. We showed that smoking in early pregnancy was associated with an increased risk of obesity and short stature in adult life. Although current rates of maternal smoking have decreased markedly since our participants were born, there remains a high prevalence of smoking amongst teenage mothers (e.g. 1 in 4 in Sweden^7^ and 1 in 3 in New Zealand^8^). Furthermore, there is also some evidence that women who smoke during pregnancy underestimate the risk to their fetus from cigarette smoking, particularly if they have fewer years of education^63,64^. Unplanned or unwanted pregnancies are both associated with a higher likelihood of maternal smoking^65^, and our findings indicate that the offspring are at risk for adverse health outcomes of maternal smoking at any time during pregnancy. As such, smoking cessation messages should target in particular younger women who may become pregnant, and medical providers should be unequivocal in expressing the risks of smoking during pregnancy for offspring health.

## Supplementary information

Supplementary Tables

## Data Availability

Data used in this study were obtained from the Swedish Medical Birth Register. While these data cannot be made publicly available, they can be accessed upon request to the Swedish National Board of Health and Welfare, pending approval by the appropriate ethics committee. Information on the Birth Register and persons to contact for queries regarding access are available in English from: www.socialstyrelsen.se/register/halsodataregister/medicinskafodelseregistret/inenglish.
