# Supplementary material for: Maternal smoking early in pregnancy is associated with increased risk of short stature and obesity in adult daughters
Source: Sci Rep. 2019 Mar 12;9:4290. doi: 10.1038/s41598-019-39006-7 (PMC6414646; doi:10.1038/s41598-019-39006-7)
Supplement: Supplementary file 1 — Supplementary Tables [file 41598_2019_39006_MOESM1_ESM.pdf]

SUPPLEMENTARY TABLES

Maternal smoking early in pregnancy is associated with increased risk of short stature and obesity in adult daughters

Sarah E Maessen[1], Fredrik Ahlsson[2], Maria Lundgren[2], Wayne S Cutfield[1,3], José G B Derraik[1,2,3*]

[1] Liggins Institute, University of Auckland, Auckland, New Zealand

[2] Department of Women's and Children's Health, Uppsala University, Uppsala, Sweden

[3] A Better Start – National Science Challenge, University of Auckland, Auckland, New Zealand

*Author for correspondence: Liggins Institute, University of Auckland, Private Bag 92019, Auckland, New Zealand; Email: j.derraik@auckland.ac.nz

Supplementary Table 1

Demographic and anthropometric data among Swedish women who were included and those who were excluded from the study.

		Excluded			Included	
		n	Mean ± SD		n	Mean ± SD
**Age (years)**		280,871	26.0 ± 4.9		22,421	26.0 ± 5.0
**Weight (kg)**		249,961	67.20 ± 12.71		22,421	67.88 ± 14.11
**Height (cm)**		262,049	167.1 ± 6.0		22,421	166.6 ± 5.9
**BMI (kg/m^2)**		245,787	24.06 ± 4.30		22,421	24.42 ± 4.77
**Birth weight (kg)**		280,391	3.42 ± 0.53		22,421	3.46 ± 0.47
**Birth length (cm)**		280,026	50.0 ± 2.3		22,362	50.0 ± 2.0
**Gestational age (weeks)**		279,582	39.7 ± 1.8		22,421	39.5 ± 1.1
**Maternal BMI (kg/m^2)**		12,830	22.30 ± 3.49		22,421	22.16 ± 3.39
**Maternal age (years)**		280,871	26.0 ± 4.1		22,421	22.2 ± 2.2

• BMI, body mass index; SD, standard deviation.

Supplementary Table 2

Anthropometric data recorded early in pregnancy (mostly 10–12 weeks) in 1991–2009 among 25,205 women who were born in Sweden in 1973–1988 according to their mothers' level of smoking early in pregnancy.

Light smoker was defined as smoking 1 to 9 cigarettes per day, and Heavier smoker as ≥10 cigarettes per day. Age data are means ± standard deviations; categorical data are n (%); other data are adjusted means and 95% confidence intervals.

	Mother Non-smoker	Mother Smoker	Mother Light Smoker	Mother Heavier Smoker
**n (%)**	14,699 (58.3%)	10,506 (41.7%)	6,116 (24.3%)	4,390 (17.4%)
**Age (years)**	26.2 ± 5.0	25.5 ± 5.1****	25.2 ± 4.9****	26.0 ± 5.2*** ††††
**Height (cm)**	167.0 (166.9-167.1)	166.1 (166.0-166.2)****	166.2 (166.1-166.3)****	166.0 (165.8-166.2)**** †
**Weight (kg)**	66.98 (66.76-67.20)	69.57 (69.32-69.81)****	69.28 (68.96-69.60)****	69.98 (69.60-70.36)**** ††
**BMI (kg/m^2)**	24.08 (24.00-24.16)	25.05 (24.97-25.14)****	24.95 (24.83-25.06)****	25.21 (25.07-25.34)**** ††
**Overweight**	3,230 (22.0%)	2,533 (24.1%)	1,473 (24.1%)	1,060 (24.1%)
**Obese**	1,519 (10.3%)	1,593 (15.2%)	868 (14.2%)	725 (16.5%)
**Overweight/obese**	4,749 (32.3%)	5,484 (39.4%)	2,341 (38.3%)	1,785 (40.7%)
**Short stature**	212 (1.4%)	238 (2.3%)	134 (2.2%)	104 (2.4%)

Overweight was defined as body mass index (BMI) ≥25 kg/m^2 and <30 kg/m^2; overweight/obese as ≥25 kg/m^2; and obese as ≥30 kg/m^2.

Short stature was defined as height more than 2 standard deviations below the population mean (i.e. <155 cm).

Adjusted data were analysed using generalized linear regression models, adjusting for birth order, age, year of birth (to account for population-wide trends), and gestational age; in addition, models on women's weight and BMI were adjusted for their current smoking habits, as well as their mother's weight or BMI, respectively; while the model on women's height was also adjusted for maternal height.

***p<0.001 and ****p<0.0001 for comparisons to daughters of Non-smokers.

†p<0.05, ††p<0.01, and ††††p<0.0001 for comparisons between daughters of Light and Heavier smokers.

Supplementary Table 3

Adjusted relative risks of short stature and obesity among 25,205 Swedish women according to their mothers’ level of smoking early in pregnancy.

Women were born in Sweden in 1973–1988, and their height and body mass index (BMI) were recorded early in pregnancy in 1991–2009 at a mean age of 25.9 years.
Light smoker was defined as smoking 1 to 9 cigarettes per day, and Heavier smoker as ≥10 cigarettes per day.

Maternal smoking levels	Short stature	*p*		Obesity	*p*
**All smokers vs Non-smokers**	1.50 (1.25, 1.81)	<0.0001		1.45 (1.36, 1.55)	<0.0001
**Light smokers vs Non-smokers**	1.48 (1.19, 1.84)	<0.001		1.37 (1.27, 1.48)	<0.0001
**Heavier smokers vs Non-smokers**	1.63 (1.29, 2.06)	<0.0001		1.57 (1.45, 1.70)	<0.0001
**Heavier smokers vs Light smokers**	1.10 (0.86, 1.41)	0.46		1.14 (1.05, 1.25)	0.003

Obesity was defined as BMI ≥30 kg/m^2, and short stature as height more than 2 standard deviations below the population mean (i.e. <155 cm).

Adjusted data were analysed using generalized linear regression models, adjusting for birth order, age, year of birth (to account for population-wide trends), and gestational age; model on obesity risk adjusted for their current smoking habits and their mother's BMI, while the model on risk of short stature was adjusted for maternal height.

Supplementary Table 4

Rates and adjusted relative risks (aRR) of obesity among 22,421 Swedish women according to their tobacco smoking habits and their mother's smoking early in pregnancy.

Women were born in Sweden in 1973–1988, and body mass index (BMI) data were recorded in 1991–2009 at a mean age of 26.0 years.

		Obesity Rate		Adjusted Relative Risk	*p*
**Mother Non-smoker**	**Daughter Non-smoker**	847 (9.6%)		reference	
	**Daughter Smoker**	495 (11.6%)		1.17 (1.05, 1.30)	0.004
**Mother Smoker**	**Daughter Non-smoker**	686 (15.2%)		reference	
	**Daughter Smoker**	726 (15.1%)		1.02 (0.92, 1.12)	0.74
**Mother Non-smoker**	**Daughter Smoker**	495 (11.6%)		reference	
**Mother Smoker**	**Daughter Smoker**	726 (15.1%)		1.35 (1.22, 1.50)	<0.0001

Obesity was defined as BMI $\geq$30 kg/m^2.

Data are n (%) or adjusted relative risk (95% confidence interval).

Data were analysed using generalized linear regression models, adjusting for birth order, age, year of birth (to account for population-wide trends), current smoking habits, and mother's BMI.
